# Real Time Imaging of Biomarkers in the Parkinson's Brain Using Mini-Implantable Biosensors. II. Pharmaceutical Therapy with Bromocriptine

**DOI:** 10.3390/ph2030236

**Published:** 2009-12-22

**Authors:** Patricia A. Broderick, Edwin H. Kolodny

**Affiliations:** 1 Department of Physiology & Pharmacology, Sophie Davis Sch. Biomed. Edu., CCNY, New York, NY 10031, USA; 2 Departments of Biology, Psychology, CUNY Grad. Sch., New York, NY 10031, USA; 3 Department of Neurology, NYU Sch. Med., Langone Med. Ctr., NYU Langone Comprehensive Epilepsy Ctr., New York, NY 10016, USA

**Keywords:** biomarkers, biosensors, Parkinson’s disease, neuromolecular imaging, electrochemistry, neurotransmitters, monoamines, dopamine, serotonin, peptides, dynorphin A, somatostatin, basal ganglia, brain, neurons, dorsal striatum, movement disorders, substantia nigra, nigrostriatal pathways

## Abstract

We used Neuromolecular Imaging (NMI) and trademarked BRODERICK PROBE^®^ mini-implantable biosensors, to selectively and separately detect neuro-transmitters *in vivo*, on line, within seconds in the dorsal striatal brain of the Parkinson’s Disease (PD) animal model. We directly compared our results derived from PD to the normal striatal brain of the non-Parkinson’s Disease (non-PD) animal. This advanced biotechnology enabled the imaging of dopamine (DA), serotonin (5-HT), homovanillic acid (HVA) a metabolite of DA, L-tryptophan (L-TP) a precursor to 5-HT and peptides, dynorphin A 1-17 (Dyn A) and somatostatin (somatostatin releasing inhibitory factor) (SRIF). Each neurotransmitter and neurochemical was imaged at a signature electroactive oxidation/half-wave potential in dorsal striatum of the PD as compared with the non-PD animal. Both endogenous and bromocriptine-treated neurochemical profiles in PD and non-PD were imaged using the same experimental paradigm and detection sensitivities. Results showed that we have found significant neurotransmitter *peptide biomarkers* in the dorsal striatal brain of endogenous and bromocriptine-treated PD animals. The *peptide biomarkers* were not imaged in dorsal striatal brain of non-PD animals, either endogenously or bromocriptine-treated. These findings provide new pharmacotherapeutic strategies for PD patients. Thus, our findings are highly applicable to the clinical treatment of PD.

## 1. Introduction

### 1.1. Background

Parkinson's Disease (PD) is prevalent, not only in the American population, but it also affects people all over the world, e.g., in Europe [1]. In fact, PD is becoming increasingly documented in developing countries that are undergoing rapid demographic changes [2]. PD exhibits multifaceted and debilitating symptoms; conventionally, though, we speak of PD as a neurodegenerative disease wherein there is a progressive impairment of movement. Some of these movement disorders are described as: (a) tremors (hands shaking while at rest), (b) rigidity (stiff, abrupt muscle movement) and (c) bradykinesia (being unable to start a particular movement, like walking). PD is caused by the loss of dopamine (DA) neurons in substantia nigra, the site for DA cell bodies in the basal ganglia, which are the motor neurons of the brain. One neuronal efferent terminal for the substantia nigra is the caudate putamen. In humans, the caudate putamen consists of two separate structures; in animals, the caudate and putamen are connected in one structure, dorsal striatum.

Research studies in animals continue to provide additional critical knowledge about PD and its ensuing pharmacologic therapies. However, surprisingly, despite all the excellent research that has been performed to-date to help ameliorate or reduce the symptoms of PD, previous experimental approaches have been unable to elucidate the precise mechanism of action of PD on DA neurons in nigrostriatal pathways. One of the limitations that previous approaches has encountered in human studies, is that brain material from early or untreated PD patients is not available and when available, there is some nigral cell death already present with the appearance of incidental Lewy bodies as well. Insofar as previous animal studies are concerned, there are little or no published studies to date that image the *in vivo* dynamic factors involved in the release of DA directly into the neuronal synapses of the brain in PD. In fact, there are little or no reports published to date, to image dynamic release of any other neurotransmitters or neurochemicals into the neuronal synapses in PD animals. 

Importantly then, real time imaging of DA in dorsal striatum is needed and indeed, neurotransmitters, other than DA, need to be imaged because other neurochemicals may play a significant role in the therapeutics of PD. The hypothesis is that DA is not the sole arbiter for the treatment of PD. The hypothesis then is two-fold: (A) that the cause and treatment of PD may involve neurotransmitters and neuromolecules other than DA and (B) that the neurochemical, biologic response to current drug therapies for PD, such as bromocriptine (Parlodel^®^) may differ in PD compared with non-PD. Therefore, in this paper, we used neuromolecular imaging (NMI) with the BRODERICK PROBE^®^ laurate biosensors to image endogenous neurochemicals in dorsal striatum of PD versus non-PD animals (Part A). We then administered the pharmaceutical, bromocriptine, intraperitoneally (ip) to PD and non-PD animals to compare neurochemical, biologic responses in dorsal striata of PD versus non-PD animals (Part B).

### 1.2. NMI and the BRODERICK PROBE^®^

**Figure 1:**
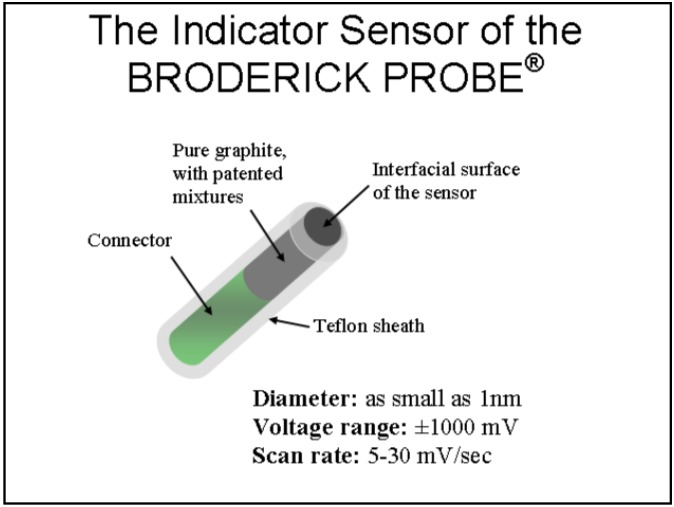
A schematic diagram of the BRODERICK PROBE^®^ biosensor. Some of the formulations are comprised of fatty acids and lipids, normal constituents of brain.

Figure 1 shows a schematic design of the mini-implantable biosensor, the BRODERICK PROBE^®^. Several formulations of the carbon-based biosensors, patented by CUNY and NYU, have been tested in controlled studies. Details for the manufacture of these biosensors, which include description of specific components of each formulation, in addition to the use, design and applications for these biosensors, are published. The NMI biotechnology, such as detector/potentiostat electrical circuits, are also published [3,4,5,6,7,8,9,10,11,12]. Patents and pending patents listed in the references herein depict novel inventive constructions and formulations of biosensors. Biosensors use electron transfer kinetics to select an image for a specific neurochemical at an electroactive oxidation/half wave potential. An electroactive signature for each neurochemical is detected in subunits of volts, amperes, dependent on the electronic circuitry chosen for use in the detector/potentiostat. In the studies presented here, a semidifferential electrical circuit was chosen because several neurotransmitters and neurochemicals can be separately imaged within one minute, each neurochemical within seconds and recordings can be repeated continuously for hours and longer periods of time, e.g., weeks and months. Using the semidifferential electrical circuit, millivolts are shown on the x axis and current in nano and picoamperes is shown on the y axis. Current is derived from electron transfer kinetics which are determined by specific biosensor properties, such as hydrophobicity and hydrophilicity of biosensor formulation within the context of interaction with specific neurotransmitters and neuromolecules. Synaptic release mechanisms are primarily studied with NMI [13].

The following formulas describe this relationship in terms of charge, electron transfer, current, diffusion layer, time, Faraday's constant, size of the indicator electrode and concentration (mass) of electroactive species: 

*Q = nFVC^o^_R _*      *i = dQ/dt*      *i = nFV dC_R,t _/ dt*

where *V* is the volume of the diffusion layer on the electrode where the measurement is being made, *n* is the number of electrons transferred, *F* is the Faraday Constant, and *C^o^* denotes initial concentration. The Cottrell equation is derived from the formulas written above and demonstrates that current i.e., charge and mass, *i.e.,* concentration, are proportional. The Cottrell equation is:

*i_t_* = *nFAC_o_D_o_*^1/2^/3.14 ^½^ t^½^

where:

i = current at time, t
n = number of electron transfers, eq/mol
F = Faraday’s constant, 96486 C/eq
A = electrode area, cm 2
C = concentration of O, mol/cm^3^
D = Diffusion coefficient of O, cm^3^/s

NMI has made several advances in the field of electrochemistry. One of these significant advances is the fact that NMI biosensors do not form gliosis, *i.e.*, scar tissue which impedes detection of neurotransmitters, causing electroactive signals to decay. This property improves the sensitivity, selectivity and operational stability of the biosensors, allowing the detection of reliable electroactive signals for long periods of time, enabling their use not only for diagnosis and treatment for PD, which we discuss here, but also for cardiac disease, e.g., acute ischemic stroke [14] and hypoxia [15,16] as well as for peripheral body disorders such as human uterine cervical cancer [4,6,7,10]. In contrast, body imaging technologies for cancer specifically that image flourescent cells also deserve mention [17,18,19,20,21]. Nonetheless, the present manuscript shows a distinct advantage over fluorescent protein imaging because unlike previous methods, The BRODERICK PROBE^®^ biosensors, have made the critical advance to clinical use, successfully, imaging neurotransmitters, neurochemicals and peptides in neocortex of epilepsy patients on line, in real time and *in vivo* during patients’ intraoperative surgery. It is important to note that the pathology reports from epilepsy patients implanted with our biosensors do not exhibit gliosis/sclerosis/scar tissue, nor do the biosensors produce bacterial growth [22,23].

### 1.3. The PD paradigm

A classical animal model of PD was used. In this animal model, a depletion of DA is produced by injecting a well-known neurotoxin, 6-hydroxydopamine (6-OHDA) into DA cell bodies, substantia nigra, during which time, DA neurons are lesioned, die, resulting in disruption of DA efferents to neuronal terminals, dorsal striatum. The end result is DA depletion in dorsal striatum. This animal model simulates what occurs in the brain of the PD patient. 

Endogenous neurochemical profiles for PD compared with non-PD were studied in Part A. In Part B, bromocriptine was administered in one study at a dose of 5 mg/kg ip (low dose) and in a second study, bromocriptine was administered at a dose of 5 mg/kg ip followed by a dose of 10 mg/kg ip (high dose).

## 2. Results and Discussion

### 2.1. *In vivo* comparison of endogenous neurochemicals in PD versus non-PD striatal brain

**Figure 2:**
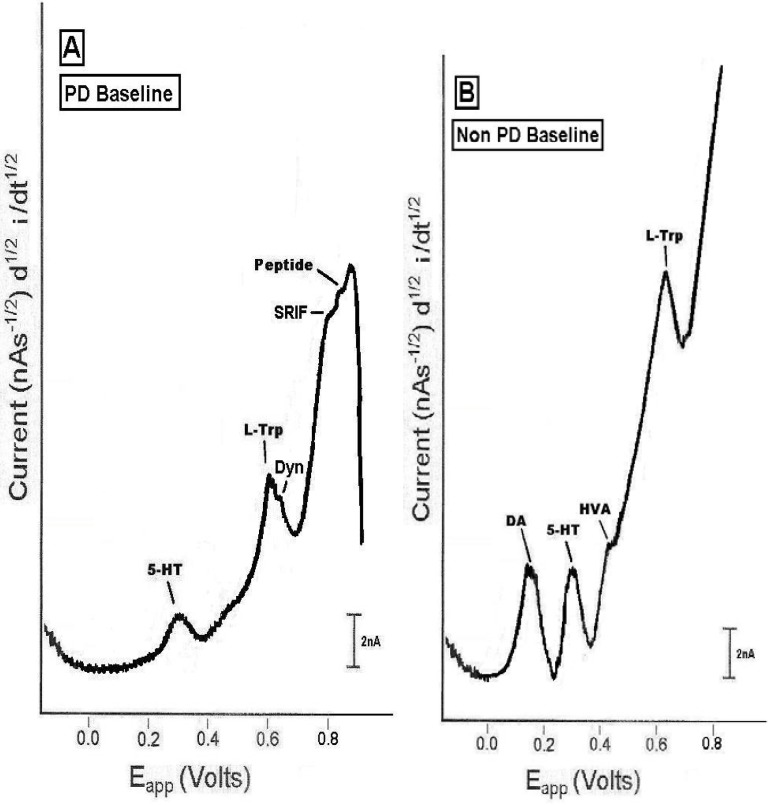
Representative NMI endogenous (baseline, control) neurochemical signature profiles in dorsal striatum of (left) the PD and (right) the non-PD animal *in vivo*, on line and in real time. Neurochemical profiles are drawn from original recordings.

Figure 2 show the results of NMI detecting endogenous neurochemicals in the PD versus the non-PD animals. Neurochemical profiles are drawn from original *in vivo* data. This is the first report of an *in vivo* neurochemical comparison between PD and non-PD in motor neurons in dorsal striatal brain. This is the first *in vivo* neurochemical profile study of PD in any neuroanatomic substrate of animal. The present data are derived from 10 NMI studies in dorsal striatum of PD versus another 10 studies in non-PD animals. 

The results showed that DA and its metabolite HVA were not imaged in striatal brain of PD. What was readily apparent in striatal brain of PD, was the selective imaging of the neurotransmitter, 5-HT and its precursor, L-TP. Moreover, two neurotransmitter peptides, Dyn A and SRIF were also separately imaged in striatal brain of PD. Repeatedly imaged to a significant degree, was a peptide at an oxidation/half-wave potential of about 0.83 V, which this laboratory is in the process of defining. *These peptide neurotransmitters were not imaged in dorsal striatal brain of non-PD animals.* In the non-PD dorsal striatum, selective electroactive signals for DA, 5-HT, HVA and L-TP were repeatedly imaged. Another observation from the data in Figure 2 is that the non-PD striatal brain endogenously exhibits higher concentrations of 5-HT and L-TP than the PD striatal brain; this interpretation derives from the Cottrell Equation which calculates that concentration of each neurochemical is directly proportional to its resultant current. 

### 2.2. *In vivo* Bromocriptine Studies in dorsal striatal brain of PD versus non-PD animals

Current therapies for PD, for the most part, depend on strategies to replace the depleted DA in the basal ganglia of PD patients. Such therapies focus on the drugs that become DA, i.e., precursors such as L-DOPA, other DA medications such as DA receptor agonists at the DA_1_, DA_2_ and DA_3_ sites, decarboxylase inhibitors, enzyme inhibitors, and DA releasers. All of these drug therapies are strategized for use in the PD patient to increase DA in dorsal striatum and substantia nigra, the motor neurons of the brain. However, whereas the appearance of movement disorders in Huntington's Disease and hemballismus may involve the amino acid neurotransmitters [25], it is unclear what neural mechanisms are associated with or cause conditions like dyskinesia (abnormal movement dysfunction) in PD. 

The pharmaceutical, bromocriptine (Parlodel^®^) was chosen for study because it is a first line therapy for PD and it is reported to act similarly to L-DOPA in the PD patient. Sometimes, it is administered as an adjunct with L-DOPA. Yet, bromocriptine is an autoreceptor agonist which autoreceptors typically are inhibitory and consequently *decrease* DA at least in the acute or initial stages of therapy. The diminution of DA in motor neurons in basal ganglia appears to be, at first glance, contradictory to classical pharmacotherapy for PD. 

Thus, the following questions were addressed:

▪ Is bromocriptine useful for PD if it may reduce DA in motor neurons?
▪ What is the mechanism of action for bromocriptine in PD patients?
▪ Can the effects of bromocriptine be biphasically dose dependent?
▪ Does bromocriptine act through other neurochemicals, neurotransmitters in PD?
▪ Is this study of bromocriptine relevant to the clinical treatment of PD?

**Figure 3:**
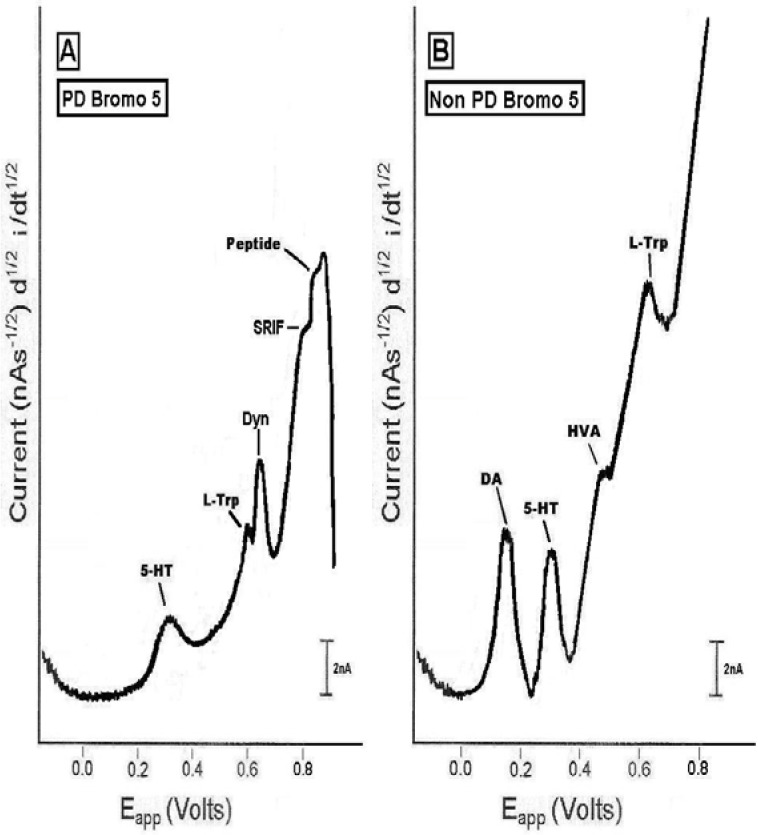
Representative NMI neurochemical signature profiles in dorsal striatum of the (left) PD and (right) the non-PD animal, after administration of the dopamine agonist, bromocriptine (5 mg/kg, ip; low dose) *in vivo*, on line and in real time. Neurochemical profiles in PD versus non-PD are drawn from original recordings.

A dose of 5 mg/kg ip was administered to the animal in the first study. In the second study, a dose of 5 mg/kg followed by a dose of 10 mg/kg, ip was administered to the animal. For animal studies, these doses are considered the low and high dose. The initial clinical dose for bromocriptine can start at 1.25 mg and then continue daily at levels of 2.5–7.5 mg daily. Nonetheless, there are reports of daily doses of bromocriptine for PD patients between 7.0 and 30 mg daily. Clinically, the low dose for PD patients has been reported to be about 30 mg daily [26], whereas the high dose has been reported to be about 52 mg daily [27]. Figure 3 show the effects of bromocriptine (5 mg/kg ip; low dose) in striatal brain of PD versus non-PD animals. 

### 2.3. *In vivo* studies of low dose bromocriptine effects on dorsal striatum in PD versus non-PD animals

This is the first report of the study of bromocriptine at any dose in the dorsal striatum or in any neuroanatomic substrate in the PD brain *in vivo*. The results from the bromocriptine administration at the low dose, showed that in the dorsal striatal brain of the PD animal, 5-HT increased about 40% above baseline, L-TP decreased about 20% from baseline, Dyn A increased about 70% above baseline and peptide at 0.83 V increased about 50% above baseline. These percentages are derived from the maximum effect of bromocriptine at the 5 mg/kg dose. The L-TP data are derived from an average of two PD studies. The Dyn A data are derived from an average of two PD studies. In addition, SRIFincreased; final percentage is in process. *It is important to note that the low dose of bromocriptine did not enable an increase in the release of DA and HVA.* Moreover, the results from the low dose of bromocriptine in the non-PD animal, showed that DA increased about 30%, 5-HT increased about 30%, HVA increased about 50% and L-TP decreased about 20%. These percentages are derived from the maximum effect of bromocriptine at the low dose. The L-TP data are derived from an average of two non-PD studies. *It is important to note that there were no peptide biomarkers imaged in the dorsal striatal brain of the non-PD animals after bromocriptine administration at the low dose*. 

### 2.4. *In vivo* studies of high dose bromocriptine effects on dorsal striatum in PD versus non-PD animals

**Figure 4:**
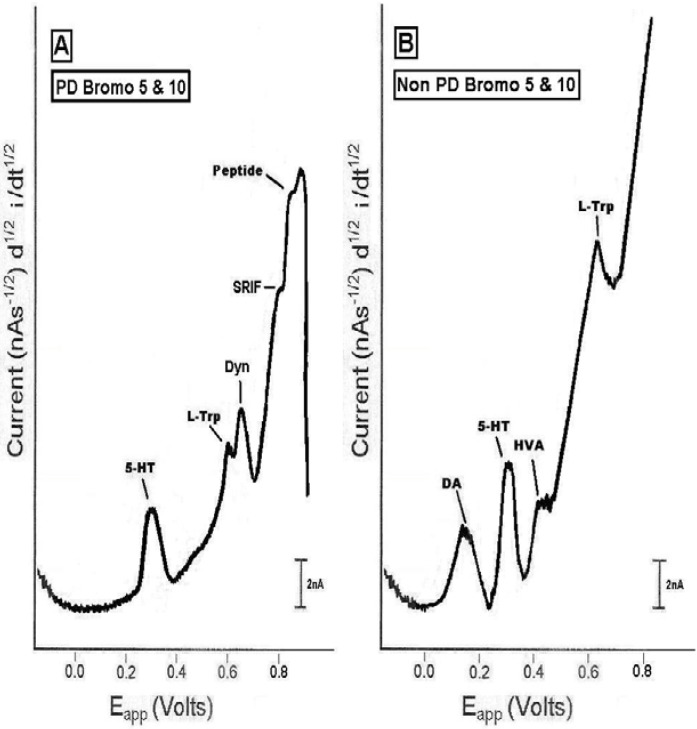
Representative NMI neurochemical signature profiles in dorsal striatum of (left) the PD and (right) the non-PD animal, after administration of the dopamine agonist, bromocriptine (5 mg/kg ip followed by 10 mg/kg ip) (high dose) *in vivo*, on line and in real time. Neurochemical profiles are drawn from original recordings.

Figure 4 show the results from the administration of bromocriptine (5 mg/kg ip followed by 10 mg/kg, ip), the high dose, in dorsal striatal brain of PD versus non-PD. The results from the administration of bromocriptine, at the high dose, showed that: in PD, 5-HT increased about 75% above baseline, L-TP decreased about 10% from baseline, Dyn A increased about 50% above baseline and peptideat 0.83 V increased about 100% above baseline. 

These percentages are derived from the maximum effect of the high dose of bromocriptine. SRIFincreased in dorsal striatum of PD; final percentage is in process. *It is important to note that bromocriptine at the high dose did not enable an increase in the concentration of DA and HVA release.* The results from the administration of bromocriptine at the high dose in dorsal striatal brain of non-PD animals, taken at maximum points, showed that DA decreased about 25% below baseline, as expected due to its autoreceptor inhibitory action and in support of microdialysis studies performed in dorsal striatal brain of non-PD animals [28]. Serotonin (5-HT) increased about 30% above baseline, HVA decreased virtually to baseline, also expectedly because as previously mentioned, HVA is a metabolite of DA; these data support those of others as studied in the non-PD animal [29]. LTP decreased about 20% from baseline. *It is important to note that the peptide biomarkers were not imaged in the dorsal striatal brain of the non-PD animal whether they were bromocriptine treated or not*. 

### 2.5. Line graphs showing the time course of endogenous effects in PD versus non-PD animals

**Figure 5:**
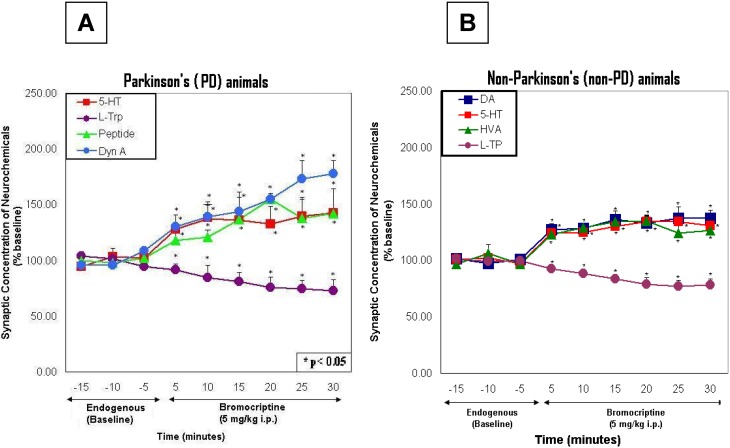
These figures show line graphs of the temporal course of neurochemical events for the PD animal (left) and the non-PD animal (right) at the 5 mg/kg dose (low dose).

Interestingly, in Figure 5, the results showed that bromocriptine, at 5 mg/kg ip, low dose, had significantly greater effects on 5-HT and L-TP in PD animals than in non-PD animals (ANOVA; p < 0.0001), even though endogenously, 5-HT and L-TP were higher in concentration in non-PD than in PD. 

### 2.6. Line graphs showing the time course of bromocriptine effects in PD versus non-PD animals

**Figure 6:**
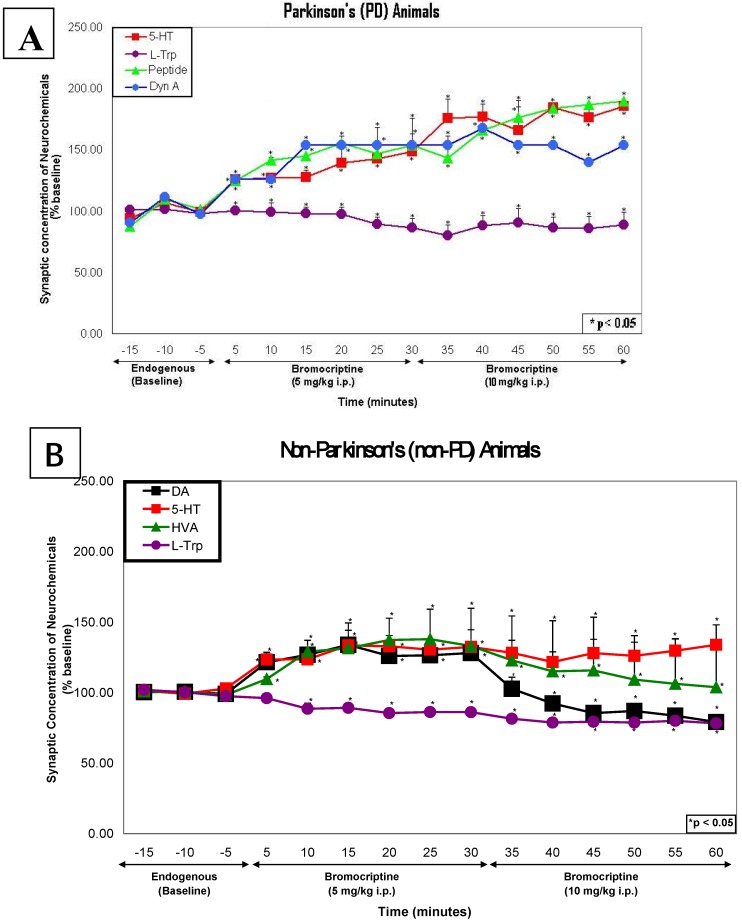
(A) This figure shows a line graph of the temporal course of neurochemical events in dorsal striatum for PD animals after the low dose followed by the high dose of bromocriptine. (B) This figure shows a line graph of the temporal course of neurochemical events in dorsal striatum of non-PD animals after administration of the high dose of bromocriptine.

In Figure 6, bromocriptine, at the high dose, also showed significantly greater effects on 5-HT and L-TP in PD animals versus non-PD animals (ANOVA; p<0.0001). It is suggested that 5-HT-ergic function may be compensating for the lack of endogenous DA and HVA in dorsal striatal brain of PD, in addition to compensating for the decreased DA and HVA biphasic effect of the high dose of bromocriptine in dorsal striatal brain of non-PD animals. 

It is important to reiterate that the high dose of bromocriptine exhibits its autoreceptor inhibitory action on DA-ergic function in dorsal striatal brain of non-PD. Both DA and HVA are decreased in non-PD animals as expected due to the biphasic dose dependent property of bromocriptine. On the other hand, the dorsal striatal brain of PD animals did not exhibit DA-ergic function at all, as expected. 

The critical pieces of information provided by these studies is (A) *Peptide biomarkers* were imaged in dorsal striatal brain of PD *in vivo*. (B) *5-HT-ergic function* appears to compensate for the DA-ergic deficiency in PD endogenously and after bromocriptine administration. (C) *Bromocriptine* did not enable the release of DA and/or HVA, i.e., DA-ergic function, at either the low or high doses of this pharmaceutical. 

However, given the caveat that the present studies are acute, single dose studies, the noted diminution in DA-ergic function, after the high dose of bromocriptine, may not occur during the chronic administration of this medication to animals and/or humans. Moreover, DA-ergic function in the basal ganglia of the brain of the PD patient may not be as dramatically reduced as was seen after substantia nigra of PD animals was lesioned with 6-OHDA. Our studies with NMI and pharmaceuticals are continuing; studies using L-DOPA are in progress.

## 3. Methods

### 3.1. Study design

In separate studies, PD (lesioned) and non-PD (non-lesioned) animals were implanted with BRODERICK PROBE^®^ biosensors in dorsal striatum under sodium pentobarbital anesthesia (50 mg/kg ip) for NMI studies. Stereotaxic coordinates were chosen according to Pellegrino et al. 1979 [24]. Surgical 6-OHDA lesions in substantia nigra of male, Sprague-Dawley *rattus norvegicus* were performed in Charles River Laboratories, NC, USA and subsequently to recovery from lesioning, animals were tested for expected PD movement defects before shipment to the CCNY Marshak Vivarium. PD and non-PD animals were purchased at the same time. Lesioned (PD) and non-lesioned (non-PD) animals (weight approximately 300 grams) were housed first in the Marshak Vivarium with required light and food requirements (12 hr dark and light cycle and food and water *ad libitum*). Protocols for PD and non-PD studies were performed with approval from the Institutional Animal Care and Use Committee (IACUC). NMI enables the same animal to be imaged as its own control; reducing animal variability and further reducing the number of animals needed for accurate statistical analysis. Recordings were taken every five minutes for a period of approximately 2 hrs.

### 3.2. Statistics

Results from PD and non-PD animals were compared by One-Way Analysis of Variance (ANOVA) with alpha level set at P=0.05. Endogenous effects and bromocriptine effects on neurotransmitters and neurochemicals in PD as compared with non-PD were statistically significant (p < 0.001). Saline effects were not significant. In Figure 5 and Figure 6, asterisks denote significance above baseline at 95% Confidence Limits (p < 0.05) and above. 

## 4. Conclusions

For the first time, neurochemical profiles for dorsal striatal brain of PD versus dorsal striatal brain of non-PD animals are reported herein *in vivo*, on line and in real time, using the advanced NMI biotechnology. Moreover, the effects of the pharmaceutical agent, routinely used to treat PD patients, bromocriptine (Parlodel^®^), was studied for its effects on neurochemical profiles for PD compared with non-PD. Critical for the treatment of PD are data reported herein that show that peptidergic neurochemistry is imaged in endogenous PD and bromocriptine-treated PD and not in endogenous non-PD whether treated with bromocriptine or not. Previous literature has shown that peptides assist the positive effects of Brain-Derived Neurotrophic Factor (BDNF) in PD [30]. *However, in this paper, unique *in vivo* data show that peptides may actually perform as biomarkers for PD.* The data suggest, in support of the work of Lu and Stoessel [31] that SRIF may help to treat PD patients. In support of the work of Henry and Brotchie [25] the present data show that antagonists of DYN A may assist in the pharmacotherapy of PD. The findings show that *DA is not the sole arbiter for the treatment of PD!* Thus, the present findings are highly applicable to the clinical treatment of PD.
